# MicroRNA‐21 protects against cardiac hypoxia/reoxygenation injury by inhibiting excessive autophagy in H9c2 cells *via* the Akt/mTOR pathway

**DOI:** 10.1111/jcmm.12990

**Published:** 2016-09-29

**Authors:** Zhouqing Huang, Shengjie Wu, Fanqi Kong, Xueli Cai, Bozhi Ye, Peiren Shan, Weijian Huang

**Affiliations:** ^1^ Department of CardiologyThe Key Lab of Cardiovascular Disease of WenzhouThe First Affiliated Hospital of Wenzhou Medical UniversityWenzhouChina

**Keywords:** miR‐21, autophagy, apoptosis, hypoxia/reoxygenation, H9c2 cells

## Abstract

MicroRNAs and autophagy play critical roles in cardiac hypoxia/reoxygenation (H/R)‐induced injury. Here, we investigated the function of miR‐21 in regulating autophagy and identified the potential molecular mechanisms involved. To determine the role of miR‐21 in regulating autophagy, H9c2 cells were divided into the following six groups: control group, H/R group, (miR‐21+ H/R) group, (miR‐21‐negative control + H/R) group, (BEZ235+ H/R) group and (miR‐21+ BEZ235+ H/R) group. The cells underwent hypoxia for 1 hr and reoxygenation for 3 hrs. Cell count kit‐8 was used to evaluate cell function and apoptosis was analysed by Western blotting. Western blotting and transmission electron microscopy were used to investigate autophagy. We found that miR‐21 expression was down‐regulated, and autophagy was remarkably increased in H9c2 cells during H/R injury. Overexpression of miR‐21 with a miR‐21 precursor significantly inhibited autophagic activity and decreased apoptosis, accompanied by the activation of the AKT/mTOR pathway. In addition, treatment with BEZ235, a novel dual Akt/mTOR inhibitor, resulted in a significant increase in autophagy and apoptosis. However, we found that miR‐21‐mediated inhibition of apoptosis and autophagy was partly independent of Akt/mTOR activation, as demonstrated in cells treated with both miR‐21 and BEZ235. We showed that miR‐21 could inhibit H/R‐induced autophagy and apoptosis, which may be at least partially mediated by the Akt/mTOR signalling pathway.

## Introduction

Ischemic heart disease has long been a leading cause of morbidity and mortality worldwide 1. Timely reperfusion after the onset of ischaemia has proven to be the most effective therapy, which can reduce cardiomyocyte apoptosis and reverse contractile dysfunction. However, it may induce another unpleasant injury, ischaemia/reperfusion injury 2, 3. Thus, the development of novel strategies to prevent myocyte death following ischaemia/reperfusion is necessary to improve the clinical outcome in patients with ischemic heart disease.

Autophagy (or, more precisely, macroautophagy) is a conserved, tightly regulated intracellular catabolic process by which mammalian cells degrade and recycle damaged and dysfunctional macromolecules and organelles 4. Enhanced levels of autophagy have been observed in the heart during both ischaemia and reperfusion 4, 5, 6. Studies have reported that autophagy has a dual, opposite role in the heart, depending on the stimulus 7. Strategies that enhance autophagy can promote survival in response to milder stress, such as brief hypoxia and low levels of oxidative stress 6, 8, whereas severe stress, such as prolonged hypoxia or subsequent reperfusion, results in excessive autophagy, which may cause cell death by triggering excessive self‐digestion of essential proteins and organelles 9, 10. The manipulation of autophagy may represent a potential future therapeutic target to protect against ischaemia/reperfusion (I/R)‐induced cardiomyocyte death and preserve cardiac function.

MicroRNAs (miRNAs) are endogenous, non‐coding, single‐stranded RNAs of approximately 22 nucleotides that can directly regulate more than 30% of the genes in a cell. miRNAs are also involved in several biological processes and are major regulators of cell differentiation, growth, proliferation and apoptosis 11, 12, 13. Recently, the roles of miR‐21 in cardiovascular biology and disease have received significant attention. miR‐21 is aberrantly expressed in many cardiovascular diseases and has been shown to play important roles in these cardiovascular disorders through both loss‐of‐function and gain‐of‐function approaches 14, 15. Studies found that miR‐21 expression was up‐regulated in the border zone of the infarcted hearts and significantly decreased in the infarcted area 16, 17. Interestingly, overexpression of miR‐21 *via* plasmid or adenovirus‐mediated gene transfer could protect against I/R‐induced cardiac cell death and decrease the myocardial infarct size, which may be mediated, at least in part, by its target genes, programmed cell death 4 (PDCD4) or phosphatase and tensin homology deleted from chromosome 10 (PTEN) 15, 16, 18. Furthermore, previous studies reported that miR‐21 could decrease autophagic activity in tumour cells by negatively regulating PTEN in tumour cells 19, 20. However, it remains unclear whether miR‐21 could influence autophagy during I/R in cardiomyocytes.

In this study, we found that miR‐21 expression was remarkably down‐regulated in H9c2 cells during hypoxia/reoxygenation (H/R) injury. We showed that miR‐21 overexpression may play a negative regulatory role in the autophagic response and protect against H/R‐induced cardiac cell death, which may be partially mediated by the Akt/mTOR signalling pathway.

## Materials and methods

### Reagents

DMEM, foetal bovine serum (FBS) and penicillin/streptomycin (pen/strep, 10,000 U/ml each) were purchased from the GIBCO Company (Life Technologies, Shanghai, China). NVP‐BEZ235 (S1009) was obtained from Selleck (Shanghai, China). The precursor of miR‐21 (cat no. BP0000850), the negative control miRNA (cat no. BP0000038) and the transfection kit (C10511‐1) were purchased from RiboBio (Guangzhou, China). The rabbit monoclonal antibody to p62 (ab109012) was obtained from Abcam (Cambridge, UK). The anti‐LC3B (cat no. 2775), Caspase‐3 (cat no. 9662), Bcl‐2 (cat no. 2870), Bax (cat no. 5023), PTEN (cat no. 9188), Akt (cat no. 9272), Phospho‐Akt (cat no. 4060), p70S6 Kinase (cat no. 2078), Phospho‐p70S6 Kinase (cat no. 9205) and GAPDH (cat no. 5174) antibodies were purchased from Cell Signaling Technology (Danvers, MA, USA). The goat anti‐rabbit secondary antibodies (cat no. 21109) used for the Western blots were obtained from Invitrogen (Carlsbad, CA, USA).

### Cell culture

The rat myocardium‐derived cell line H9c2 was obtained from the American Type Culture Collection (ATCC, Rockville, MD, USA) and maintained in DMEM containing 4500 mg/l glucose, 10% FBS, 10 mM HEPES (Sigma‐Aldrich, St Louis, MO, USA) and a 1% penicillin/streptomycin solution at 37°C in a 5% CO_2_ incubator (Thermo, Waltham, MA, USA). We induced H/R in H9c2 cells to simulate I/R injury. First, substrate‐free medium (serum‐free, glucose‐free) was pre‐treated under hypoxic conditions (1% O_2_, 95% N_2_ and 5% CO_2_) at 37°C for 2 hrs to reach a hypoxic status. Then, the H9c2 cells were cultured in the hypoxic substrate‐free medium saturated under the hypoxic conditions at 37°C for 1 hr. Simulated hypoxia was followed by a simulated reoxygenation period, during which the cells were exposed to the normoxic culture medium at 37°C for 3 hrs. The cells in the normal control groups were exposed to the normoxic conditions for 4 hrs.

### MicroRNA transfection

Cells in exponential phase of growth were plated in six‐well plates at 2 × 10^5^ cells/plate and cultured overnight. Then, the cells were transfected with the miR‐21 precursor (50 nM) or a negative control RNA (50 nM) using riboFECT^™^ CP Reagent and Buffer (RiboBio, Guangzhou, China), according to the manufacturer's protocol. Briefly, 6.25 μl of the miRNAs was diluted with 150 μl riboFECT^™^ CP buffer at 37°C for 10 min. The diluent was mixed with 15 μl riboFECT^™^ CP Reagent and incubated for 10 min. at 37°C. Then, the riboFECT^™^CP‐miRNA mixture was added to the cells together with 1.8 ml DMEM and incubated at 37°C for 48 hrs.

### CCK‐8 assay

Cell viability was assessed with the cell count kit‐8 (CCK‐8; Beyotime Biotech, Jiangsu, China), according to the manufacturer's instructions. H9c2 cells were plated in 96‐well plates at 2 × 10^3^ cells/plate. When the treatments were completed, the culture medium was replaced with 100 μl of CCK‐8 solution (containing 90 μl of serum‐free DMEM and 10 μl of CCK‐8 reagent). We measured the colour intensity with an enzyme‐mark analyser (Pulangxin, Beijing China) at a wavelength of 450 nm.

### Real‐time quantitative PCR

Total RNA was extracted from the H9c2 cells using Trizol reagent according to the manufacturer's protocol (Invitrogen Life Technologies). One microgram of total RNA from each sample was used to generate cDNAs using the RevertAid^™^ First Strand cDNA Synthesis Kit (#K1622; Thermo) with a special stem‐loop primer for miRNAs. Based on the miR‐21 sequence, a stem‐loop RT primer was designed with the following sequence: 5′‐GTCGTATCCAGTGCAGGGTCCGAGGTATTCGCACTGG ATACGACTCAACA‐3′. Then, qRT‐PCR was performed to quantify the miR‐21 expression level with SYBR Green PCR Master Mix (#K0223; Thermo), according to the manufacturer's instructions. U6 was used as an internal control. The forward and reverse primers sequences for miR‐21 were as follows: 5′‐GCCGCTAGCTTATCAGACTGATGT‐3′ and 5′‐GTGCAGGGTCCGAGGT‐3′. The forward and reverse primers sequences for U6 were as follows: 5′‐CTCGCTTCG GCAGCACA‐3′ and 5′‐AACGCTTCAC GAATTTGCGT‐3′. The qRT‐PCR was performed on ABI 7300 thermocycler (Applied Biosystems, Foster City, CA, USA). The 2^−ΔΔCT^ relative quantification method was applied.

### Protein isolation and Western blotting analysis

The cells were washed with PBS, lysed in lysed buffer on ice and then cleared by centrifugation at 12,000 × g for 10 min at 4°C. Equal amounts (20 μg) of cell lysates were resolved on 10% or 15% SDS‐PAGE gels and transferred to PVDF membranes. These membranes were blocked in 5% non‐fat dry milk in TBST with 0.1% Tween‐20 at room temperature for 2 hrs, followed by incubation with the primary antibodies overnight at 4°C. The membranes were washed and incubated with a horseradish peroxidase‐conjugated secondary antibody for 1 hr at room temperature. The antibody complexes were visualized and quantified using an enhanced chemiluminescence–Western blotting detection system (Tanon, Shanghai, China). The protein expression levels were quantified by relative densitometry and normalized to GAPDH as an internal control.

### Transmission electron microscopy

Autophagic vacuoles are defined as structures enclosed by a double membrane and filled with degenerated organelles, which can be detected by transmission electron microscopy 21. In this study, the cells were washed with 0.1 M cacodylate buffer, fixed in 2.5% glutaraldehyde in 0.1 M cacodylate buffer for 1 hr and then post‐fixed in 1% osmium tetroxide buffer. After dehydration in serially diluted ethanol solutions, the cells were embedded in an epoxy resin. Ultrathin (~70 nm) sections were cut and placed on the grids. The sections on the grids were double stained with uranyl acetate and lead citrate before being examined under a transmission electron microscope (JEM‐1230; JEOL, Tokyo, Japan). In addition, the density of autophagic vacuoles was calculated using the following formula: vacuoles/number of cells.

### Statistical analysis

All experiments were performed in triplicate at a minimum. The data were presented as the means ± S.D. and analysed by one‐way anova, followed by all pair‐wise multiple comparison procedures using Bonferroni's test. A *P*‐value of <0.05 was considered statistically significant. The statistical analysis was performed with SPSS version 20.0 (SPSS, Chicago, IL, USA).

## Results

### miR‐21 expression in H9c2 cells after H/R stimulation

**Figure 1:**
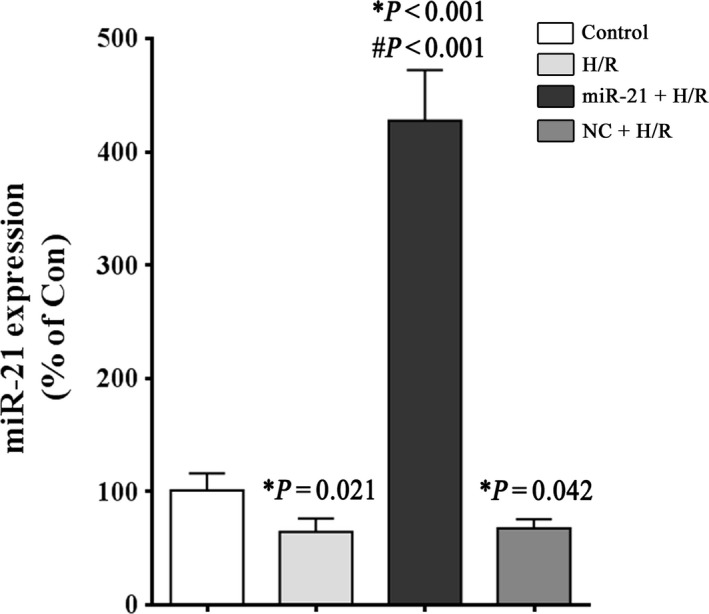
miR‐21 expression in H9c2 cells after H/R stimulation. H9c2 cells were exposed to 1 hr of hypoxia, followed by 3 hrs of reoxygenation. The control groups were not subjected to hypoxia/reoxygenation. At the end of the treatment, miR‐21 expression was analysed by Real‐time RT‐PCR, as described in the ‘Material and methods’. A small nuclear RNA (RNU66) was used as an internal control. *P versus the control group; #*P versus* the H/R group.

First, we observed decreased endogenous miR‐21 expression in H9c2 cells after H/R injury by qRT‐PCR. Compared to the control group, miR‐21 expression in the H/R group was 64.3%. These data indicate that miR‐21 down‐regulation may be related to cardiomyocyte H/R injury. To explore the role of miRNA‐21 in H/R‐induced autophagy and apoptosis, we increased miR‐21 expression using the miR‐21 precursor and a negative control (NC). As shown in Figure 1, cells transfected with the miR‐21 precursor showed an approximately sevenfold increase in miR‐21 expression compared to the H/R group. There was no significant difference in miR‐21 expression between the NC group and H/R group.

### miR‐21 up‐regulation alleviates autophagy induced by H/R injury

**Figure 2:**
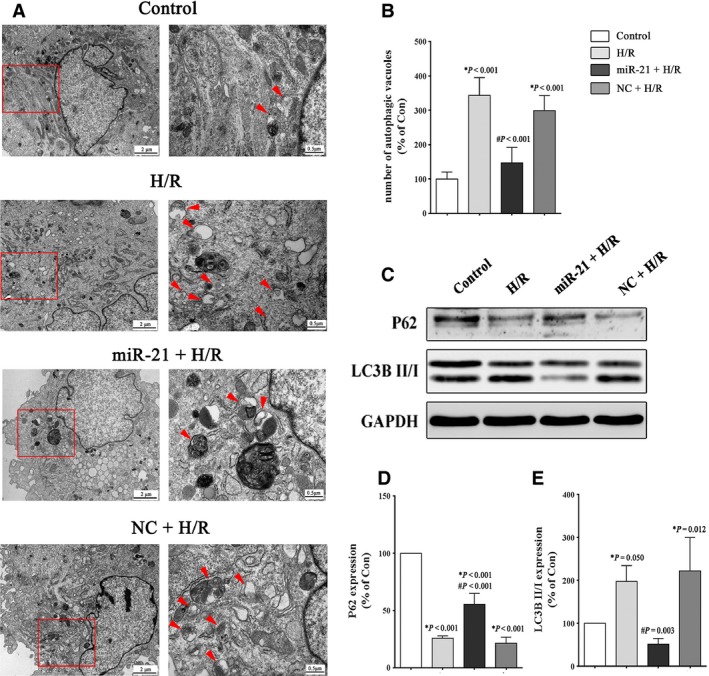
Effect of miR‐21 on the autophagic activity of H9c2 cells after H/R stimulation. H9c2 cells were exposed to 1 hr of hypoxia, followed by 3 hrs of reoxygenation. The control groups were not subjected to hypoxia/reoxygenation. (**A**) Representative TEM pictures of the intracellular ultrastructures of H9c2 cells. The arrows indicate autophagic vacuoles. (**B**) Quantification of the number of autophagic vacuoles and autophagosomes. Ten cells from each group were examined, and the average number is shown in the histogram. (**C**–**E**) Representative Western blot and quantitative evaluation of the expression of P62 and LC3B. *P versus the control group; #*P versus* the H/R group.

Intracellular autophagic vacuoles and autophagosomes in the H9c2 cells were visualized and quantified by TEM. The formation of autophagic vacuoles and autophagosomes in the H9c2 cells was increased after H/R exposure, whereas pre‐treatment with the miR‐21 precursor dramatically reduced autophagy (Fig. 2A). As shown in Figure 2B, the number of autophagic vacuoles and autophagosomes in the H/R group was increased 2.4‐fold compared to the control group (*P* < 0.001), and the miR‐21 precursor decreased this increase to approximately 1.5‐fold (*P* < 0.001) in the (miR‐21+ H/R) group. Figure 2C and E showed that miR‐21 up‐regulation resulted in a decrease in the ratio of LC3‐II/LC3‐I compared to the H/R group (51.2 ± 13.3% *versus* 197.3 ± 37.0%, *P* = 0.003), which is a reliable indicator of autophagy 21. A decrease in the levels of the ubiquitin‐binding protein p62 occurs when autophagy is activated because p62 is an autophagy substrate 21. We assayed the p62 protein levels following the miR‐21 precursor treatment and observed an approximate 1.5‐fold increase in p62 protein expression in H9c2 cells compared to the H/R group (Fig. 2C and D).

### miR‐21 up‐regulation alleviates apoptosis induced by H/R injury

**Figure 3:**
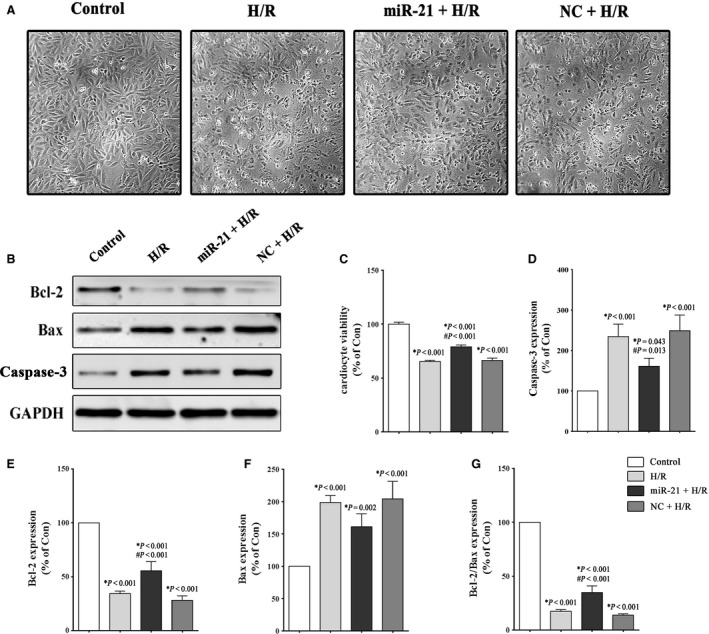
Effect of miR‐21 on H9c2 cell apoptosis after H/R stimulation. The H9c2 cells were exposed to 1 hr of hypoxia, followed by 3 hrs of reoxygenation. The control groups were not subjected to hypoxia/reoxygenation. (**A**) Microscopic cellular morphology. (**B**,** D**–**G**) Representative Western blot and quantitative evaluation of the expression of Caspase‐3, Bcl‐2 and Bax. (**C**) The viability of H9c2 cells was detected using the CCK‐8 assay. *P versus the control group; #*P versus* the H/R group.

Figure 3A depicts the effects of simulated H/R on the H9c2 cellular morphology observed by microscopic examination. We observed that cell viability was decreased in the H/R group, whereas cells treated with the miR‐21 precursor exhibited an amelioration of the cell loss induced by H/R injury. As shown in Figure 3C, the (miR‐21+ H/R) group had a higher percentage of viable cells compared to the H/R group (78.9 ± 17.6% *versus* 61.2 ± 12.6%, *P* < 0.001). The results of the CCK‐8 assay confirmed the anti‐apoptotic role of miR‐21. In addition, the apoptosis‐related proteins Caspase‐3, Bcl‐2 and Bax were detected by Western blotting. As shown in Figure 3D, the H/R group showed an approximate 2.3‐fold increase in Caspase‐3 expression compared with the control group, which was reduced to 1.6‐fold when the cells were treated with the miR‐21 precursor (*P* < 0.001). In addition, H/R stimulation increased the expression of Bax and down‐regulated the expression of Bcl‐2. Treatment with the miR‐21 precursor markedly reversed the effect on the expression levels of Bcl‐2 and Bax in the cells exposed to H/R. Figure 3G shows a higher ratio of Bcl‐2 to Bax in the (miR‐21+ H/R) group than in the H/R group (34.7 ± 6.3% *versus* 17.3 ± 1.6%, *P* < 0.001).

### miR‐21 can signal through the PTEN/Akt/mTOR axis in the H9c2 cells

**Figure 4:**
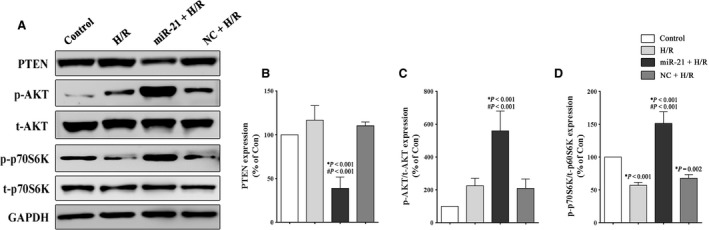
Effect of miR‐21 on the Akt/mTOR signalling pathway in H9c2 cells. The H9c2 cells were exposed to 1 hr of hypoxia, followed by 3 hrs of reoxygenation. The control groups were not subjected to hypoxia/reoxygenation. (**A**–**D**) Representative Western blot and quantitative evaluation of the expression of PTEN, phospho‐Akt, total‐Akt, phospho‐p70S6K and total‐p70S6K. *P versus the control group; #*P versus* the H/R group.

We sought to examine the significance of miR‐21‐mediated regulation of the PTEN/Akt/mTOR signalling pathway in H9c2 cells during H/R injury. Our results showed that H/R stimulation induced a mild increase in the expression of the apoptosis‐related protein PTEN, but the difference was not statistically significant. Moreover, we observed that p‐Akt is activated and p‐p70S6K is inhibited after H/R stimulation (Fig. 4A–D). Furthermore, as shown in Figure 4C and D, a significant up‐regulation of the p‐Akt/t‐Akt (560.2 ± 120.2% *versus* 218.2 ± 50.1%, *P* < 0.001) and p‐p70S6K/t‐p70S6K (151.3 ± 17.9% *versus* 57.1 ± 4.6%, *P* < 0.001) levels was induced by the miR‐21 precursor compared to the H/R group. In summary, the results implied that miR‐21 can signal through the PTEN/Akt/mTOR axis in the H9c2 cells during H/R injury.

### Inhibiting the Akt/mTOR axis partially abrogates the miR‐21‐mediated protection during H/R injury

**Figure 5:**
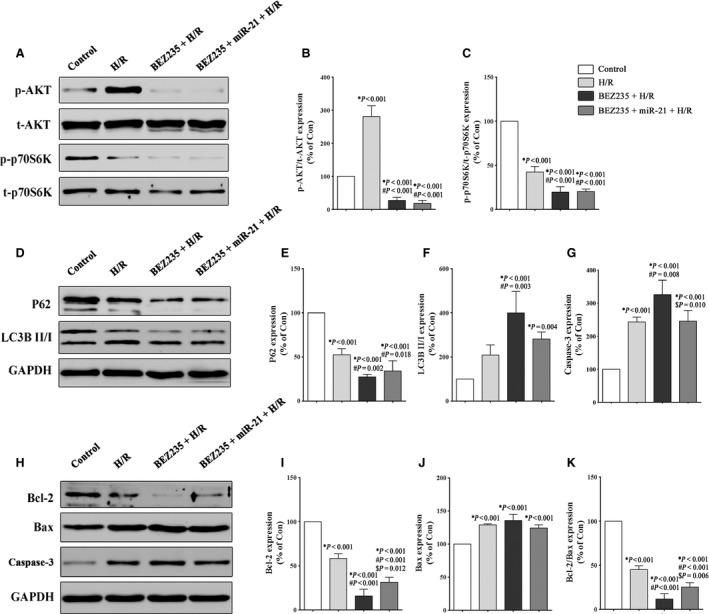
Inhibiting the Akt/mTOR signalling pathway partially abrogates miR‐21‐mediated protection during H/R injury. The H9c2 cells were exposed to 1 hr of hypoxia, followed by 3 hrs of reoxygenation. The control groups were not subjected to hypoxia/reoxygenation. The cells were treated with BEZ235 (100 nmol/l) for 2 hrs before being transfected with the miR‐21 precursor. (**A**–**C**) Representative Western blot and quantitative evaluation of the expression of phospho‐Akt, total‐Akt, phospho‐p70S6K and total‐p70S6K. (**D**–**F**) Representative Western blot and quantitative evaluation of the expression of the autophagy‐related proteins p62 and LC3B. (**G**–**K**) Representative Western blot and quantitative evaluation of the expression of the apoptosis‐related proteins Caspase‐3, Bcl‐2 and Bax. *P versus the control group; #*P versus* the H/R group; $*P versus* the (BEZ235+ HR) group.

We inhibited the Akt/mTOR signalling pathway using NVP‐BEZ235, a novel dual Akt/mTOR inhibitor, to determine whether there was a direct link between the miR‐21‐induced activation of the PTEN/Akt/mTOR signalling pathway and apoptosis and autophagy. The data presented in Figure 5B and C show a significant inhibition of the Akt/mTOR axis in the BEZ235‐treated cells. Figure 5E and F show that BEZ235 was able to enhance autophagy by down‐regulating p62 (27.3 ± 3.0% *versus* 52.4%, *P* = 0.002) and up‐regulating the LC3B II‐to‐LC3B I ratio (399.1 ± 98.4% *versus* 208 ± 46.2%, *P* = 0.003). Compared to the (BEZ235+ H/R) group, the (BEZ235+ miR‐21+ H/R) group displayed a higher expression of p62 (34.1 ± 11.6% *versus* 27.3 ± 3.0%, *P* = 1.000) and a reduced LC3B II‐to‐LC3B I ratio (281.6 ± 31.3% *versus* 399.1 ± 98.4%, *P* = 0.074); however, the differences were not statistically significant. Moreover, compared to the H/R group, the BEZ235 treatment led to an increase in apoptosis, with a higher expression of Caspase‐3 and Bax and a lower expression of Bcl‐2. Meanwhile, as shown in Figure 5G and K, the expression of Caspase‐3 and the Bcl‐2 to Bax ratio showed that treatment with the miR‐21 precursor in the (BEZ235+ miR‐21+ H/R) group could decrease apoptosis compared to the (BEZ235+ H/R) group (245.3 ± 32.6% *versus* 325.9 ± 43.8%, *P* = 0.010; 25.2 ± 5.0% *versus* 11.8 ± 5.9%, *P* = 0.006, respectively). Altogether, these results suggested that miR‐21 could inhibit apoptosis and autophagy induced by H/R stimulation partially through the activation of the PTEN/Akt/mTOR signalling pathway.

## Discussion

To our knowledge, this is the first study to show that miR‐21 up‐regulation could inhibit the excessive autophagy induced by H/R stimulation in H9c2 cells, which plays an important role in protecting the cells from cardiac H/R injury. The results presented in this study indicate that the mechanism by which miR‐21 regulates autophagy may be to target the Akt/mTOR signalling pathway.

Autophagy is a dynamic and highly regulated process of self‐digestion. It is becoming increasingly recognized that autophagy is an intricate process with multiple biological aspects that is associated with a number of diseases, such as heart disease 22, 23. As the first studies described a role for autophagy in the heart 24, considerable efforts have been focused on the role of autophagy in cardiovascular disease. Under basal conditions, autophagy is cytoprotective and promotes normal function in the heart. However, stimulated autophagy has been implicated in several types of cardiomyopathy 25. Autophagy is anti‐apoptotic and contributes to cellular recovery during brief myocardial ischaemia, but the subsequent reperfusion could cause excessive autophagy, which is considered cytotoxic and termed as class II programmed or autophagic cell death 6, 7, 8, 26. Our previous studies showed that inhibiting excessive autophagy could significantly decrease the infarct size and improve cardiac function after I/R‐induced myocardial injury 27, 28. A similar result was observed *in vitro* in this study.

miRNA molecules play a pivotal role in virtually all cellular functions, including apoptosis, proliferation, migration and differentiation 12, 13. The first report on the role of miRNAs in regulating autophagy was published in 2009 by Zhu *et al*. 29. The authors reported that miR‐30a could modulate autophagy by inhibiting Beclin 1 expression, and this effect is mediated *via* the miR‐30a consensus sequences contained in the 3′‐UTR of Beclin 1. Subsequently, a number of miRNAs were proven to be able to modulate autophagy by targeting their mRNAs or other signalling pathways 30, 31. Recently, Gwak *et al*. showed that silencing of miR‐21 could enhance autophagy and increase radiosensitivity in malignant glioma cell lines through inhibition of the PTEN/Akt pathway 19. Similarly, another study identified that transfection of miR‐21 mimics in hepatocellular carcinoma cells can restore sorafenib resistance by inhibiting autophagy 32. In this study, we showed that miR‐21 up‐regulation played a negative regulatory role in autophagy, which may be at least partially mediated by the PTEN/Akt/mTOR signalling pathway.

The phosphoinositide‐3‐kinase (PI3K)/Akt/mTOR pathway is known to play a key role in numerous cellular functions, including proliferation, adhesion, migration and survival 33. mTOR(p70S6K), a central regulator of cell growth, was shown to negatively regulate autophagic activity 34. The mechanisms by which this pathway is activated include the loss of PTEN function, RAS mutation and overexpression of growth factor receptors 33, 35. PTEN, a known tumour suppressor, inhibits the PI3K pathway and prevents Akt activation 36. mPTEN is the currently identified target gene of miR‐21 15. We showed that miR‐21 up‐regulation inhibited PTEN expression and activated the PI3K/Akt/mTOR pathway, resulting in down‐regulation of autophagy. However, we showed that miR‐21 decreases autophagic activity when the PI3K/Akt/mTOR pathway is blocked by NVP‐BEZ235, a novel dual Akt/mTOR inhibitor, which indicated that other potential signalling pathways may be regulated by miR‐21. miR‐21 has also been shown to target Bcl‐2 *via* a direct interaction as it binds to the Bcl‐2 mRNA and increases Bcl‐2 expression 37. Beclin 1, a Bcl‐2 homology 3 (BH3) domain‐only protein, is an essential initiator of autophagy, which can be inhibited by Bcl‐2 *via* its BH3 domain 38. Seca *et al*. showed that treatment with an anti‐miR‐21 antibody induces an increase in the levels of the autophagy‐related proteins Beclin‐1, Vps34 and LC3‐II by decreasing Bcl‐2 expression 39. Our results also showed a negative interaction between Bcl‐2 and autophagic activity. Recently, growing evidence showed that members of the RAB small GTPase protein family, well‐known regulators of membrane trafficking and fusion may play key roles in the regulation of the autophagic process 40. A study of renal ischaemia/reperfusion reported that miR‐21 directly targets Rab11a and inhibits autophagic activity, which suggested a new potential signalling pathway by which miR‐21 modulates autophagy 41.

In this study, we only aimed to investigate the effect and mechanism of miR‐21 up‐regulation in cell‐based experiments *in vitro* as a preliminary exploration. However, animal studies on miR‐21 up‐regulation and knockout or inhibition could better reveal the correlation among miR‐21, autophagy and apoptosis and, thus, should be performed in our further studies.

In summary, our data suggest that miR‐21 up‐regulation is able to inhibit the excessive autophagic activity and alleviate H9c2 cell apoptosis induced by H/R, and the potential mechanism is involved in the regulation of the PTEN/Akt/mTOR signalling pathway.

## Conflicts of interest

The authors report that there are no conflicts of interest.
